# Clinical Evaluation of the cobas SARS-CoV-2 Test and a Diagnostic Platform Switch during 48 Hours in the Midst of the COVID-19 Pandemic

**DOI:** 10.1128/JCM.00599-20

**Published:** 2020-05-26

**Authors:** Mario Poljak, Miša Korva, Nataša Knap Gašper, Kristina Fujs Komloš, Martin Sagadin, Tina Uršič, Tatjana Avšič Županc, Miroslav Petrovec

**Affiliations:** a Institute of Microbiology and Immunology, Faculty of Medicine, University of Ljubljana, Ljubljana, Slovenia; Boston Children’s Hospital

**Keywords:** SARS-CoV-2, COVID-19, cobas, cobas 6800

## Abstract

Laboratories are currently witnessing extraordinary demand globally for sampling devices, reagents, consumables, and diagnostic instruments needed for timely diagnosis of severe acute respiratory syndrome coronavirus 2 (SARS-CoV-2) infection. To meet diagnostic needs as the pandemic grows, the U.S. Food and Drug Administration (FDA) recently granted several commercial SARS-CoV-2 tests Emergency Use Authorization (EUA), but manufacturer-independent evaluation data are scarce. We performed the first manufacturer-independent evaluation of the fully automated sample-to-result two-target test cobas 6800 SARS-CoV-2 (cobas) (Roche Molecular Systems, Branchburg, NJ), which received U.S. FDA EUA on 12 March 2020. The comparator was a standardized 3-h SARS-CoV-2 protocol, consisting of RNA extraction using an automated portable instrument, followed by a two-target reverse transcription real-time PCR (RT-PCR), which our laboratory has routinely used since January 2020 [V. M. Corman, O. Landt, M. Kaiser, R. Molenkamp, et al., Euro Surveill 25(3):pii=2000045, 2020, https://doi.org/10.2807/1560-7917.ES.2020.25.3.2000045]. cobas and the comparator showed overall agreement of 98.1% and a kappa value of 0.95 on an in-house validation panel consisting of 217 well-characterized retrospective samples. Immediate prospective head-to-head comparative evaluation followed on 502 samples, and the diagnostic approaches showed overall agreement of 99.6% and a kappa value of 0.98. A good correlation (*r*^2^ = 0.96) between cycle threshold values for SARS-CoV-2-specific targets obtained by cobas and the comparator was observed. Our results showed that cobas is a reliable assay for qualitative detection of SARS-CoV-2 in nasopharyngeal swab samples collected in the Universal Transport Medium System (UTM-RT) (Copan, Brescia, Italy). Under the extraordinary circumstances that laboratories are facing worldwide, a safe diagnostic platform switch is feasible in only 48 h and in the midst of the COVID-19 pandemic if carefully planned and executed.

## Abstract (precis)

Laboratories are currently witnessing extraordinary demand globally for sampling devices, reagents, consumables, and diagnostic instruments needed for timely diagnosis of severe acute respiratory syndrome coronavirus 2 (SARS-CoV-2) infection. To meet diagnostic needs as the pandemic grows, the U.S. Food and Drug Administration (FDA) recently granted several commercial SARS-CoV-2 tests Emergency Use Authorization (EUA), but manufacturer-independent evaluation data are scarce. We performed the first manufacturer-independent evaluation of the fully automated sample-to-result two-target test cobas 6800 SARS-CoV-2 (cobas) (Roche Molecular Systems, Branchburg, NJ), which received U.

## INTRODUCTION

Coronavirus disease 2019 (COVID-19), a severe acute respiratory syndrome first linked to a seafood market in Wuhan (Hubei, China) in December 2019 (1, 2), has become a major public health concern all over the world. The pandemic is rapidly evolving, and as of 8 April 2020, there have been 1,447,466 laboratory-confirmed cases of COVID-19 with 83,471 deaths. The causative agent of COVID-19 is a severe acute respiratory syndrome coronavirus 2 (SARS-CoV-2; previously known as 2019-nCoV) (2, 3), which is genetically related to but distinct from two other coronaviruses responsible for large-scale outbreaks in the past: severe acute respiratory syndrome coronavirus (SARS-CoV) (about 79% sequence identity) and Middle East respiratory syndrome coronavirus (MERS-CoV) (about 50%) (2, 3).

As evidenced in previous coronavirus epidemics caused by SARS-CoV and MERS-CoV (4, 5), highly reliable laboratory diagnostics for COVID-19 are essential for case identification, patient management, and contact tracing. Due to the current limitations of other laboratory approaches (viral culture, antigen testing, and serology), reverse transcription real-time PCR (RT-PCR) remains the most suitable laboratory diagnostic test for COVID-19 (6–8). The rapid availability of the complete genome of SARS-CoV-2 early in the pandemic (9, 10) facilitated the development of standardized laboratory PCR protocols for COVID-19 in January 2020 (7, 8, 10–12), followed by the launch of a range of commercial SARS-CoV-2 PCR-based assays in the last 3 months. Despite the fact that the U.S. Food and Drug Administration (FDA) granted several commercial SARS-CoV-2 amplification assays Emergency Use Authorization (EUA), as of 29 March 2020, no manufacturer-independent evaluation data for any commercial SARS-CoV-2 assay with U.S. FDA EUA is available in the peer-reviewed literature.

Here, we present the results of the first manufacturer-independent evaluation of the fully automated sample-to-result two-target test cobas 6800 SARS-CoV-2 (cobas) (Roche Molecular Systems, Branchburg, NJ, USA), which received U.S. FDA EUA on 12 March 2020. The performance of the cobas SARS-CoV-2 test was first evaluated on a well-characterized in-house validation panel consisting of 217 samples. The comparator was a standardized 3-h SARS-CoV-2 detection protocol, consisting of RNA extraction using an automated portable instrument, followed by RT-PCR targeting the envelope (E) and RNA-dependent RNA polymerase (RdRp) coronavirus genes (11). The comparator protocol, which has been in routine use in our laboratory since January 2020, was originally developed by Christian Drosten’s team (Charité Hospital, Berlin, Germany) (11) and is recommended by the World Health Organization (WHO) (13, 14). The protocol is currently considered the gold standard for SARS-CoV-2 detection in Europe (6, 15). The initial evaluation on the validation panel was followed by immediate prospective head-to-head comparison on 502 routinely collected clinical samples against the same comparator. The entire cobas evaluation procedure followed by a successful diagnostic platform switch lasted only 48 h and was performed in the midst of the COVID-19 pandemic.

## MATERIALS AND METHODS

### In-house validation panel and comparator protocol.

The well-characterized in-house validation panel consisted of 217 undiluted 600-μl aliquots of nasopharyngeal (*n* = 211) or combined nasopharyngeal/oropharyngeal swabs (*n* = 6) collected in a Universal Transport Medium System (UTM-RT) (Copan, Brescia, Italy). The panel was designed to test sensitivity on a number of SARS-CoV-2-positive samples, with a wide range of viral loads and specificity using SARS-CoV-2-negative samples, as well as samples positive for other respiratory viruses related and not related to SARS-CoV-2. The clinical samples used in the validation panel were collected from 217 individuals referred for COVID-19 testing from 7 March 2020 to 23 March 2020.

All samples in the validation panel were routinely tested for the presence of SARS-CoV-2 immediately upon arrival in the laboratory using a standardized 3-h protocol implemented in our laboratory in early January 2020. Briefly, swabs were vortexed for 1 min at maximum speed followed by automatic nucleic acid extraction from 200 μl of UTM-RT conducted on a MagNa Pure Compact instrument (Roche Applied Science, Mannheim, Germany) using a MagNA Pure Compact Nucleic Acid isolation kit I (Roche), following the manufacturer’s instructions. Equine arteritis virus, a positive-sense single-stranded RNA virus, was added to all clinical specimens prior to RNA extraction and served as an internal extraction and amplification control (16). For detection of SARS-CoV-2, two-target RT-PCRs (SARS-CoV-2 specific and pan-sarbecovirus) were performed using previously described commercially available primers and 6-carboxyfluorescein (FAM)-labeled hydrolysis probes (11). LightMix Modular SARS and Wuhan CoV E-gene kit (Tib-Molbol, Berlin, Germany) amplifying a 76-bp-long fragment from a conserved region in the E gene (pan-sarbecovirus target) and LightMix Modular Wuhan CoV RdRp gene kit (Tib-Molbol) amplifying a 100-bp-long fragment from a conserved region of the RNA-dependent RNA polymerase (RdRp) gene (a SARS-CoV-2 specific target) was used in combination with TaqMan Fast Virus 1-Step MasterMix (Thermo Fisher Scientific, Grand Island, NY, USA). RT-PCR was performed on the StepOnePlus real-time PCR system (Applied Biosystems, Thermo Fisher Scientific) following recommended cycling conditions: reverse transcription at 50°C for 5 min and 95°C for 20 s, followed by 45 cycles of PCR, with 1 cycle consisting of 95°C for 3 s and 60°C for 30 s (11). Cycle threshold (*C_T_*) values for the LightMix two-target RT-PCR (LightMix) were always set at 0.1 normalized reporter dye intensity (delta Rn). *C_T_* values above 37.0 were considered negative. The tested sample was considered SARS-CoV-2 positive if LightMix showed positive results for either both the E (pan-sarbecovirus target) and RdRp genes (SARS-CoV-2-specific target) or the RdRp gene only. In the case of positivity for the E gene only, the result was reported as SARS-CoV-2 presumptive positive, and a follow-up sample was requested. The SARS-CoV-2 positive-control panel needed for initial assay implementation was obtained from European Virus Archive Global (EVAg) (www.european-virus-archive.com).

All samples included in the validation panel that tested negative using LightMix were additionally tested for the presence of other common respiratory viruses using the commercially available Respiratory Viruses 16-well assay (AusDagnostics, Mascot, Australia), as previously described (17, 18). The assay utilizes a multiplex tandem PCR (MT-PCR) for enrichment of targets followed by amplification of targeted DNA and/or RNA. The viruses targeted are influenza virus A (H1, H3, H5, and H7), influenza virus B (Yamagata and Victoria lineages), respiratory syncytial virus (types A and B), rhinovirus (types A, B, and C), enterovirus (types A, B, C, and D), human bocavirus 1, human parainfluenza virus (types 1 to 4), human parechovirus (types 1 to 8), human adenovirus (groups B, C, and E and some from groups A and D), human coronavirus (229E, HKU-1, NL63, and OC43), and human metapneumovirus (types A and B). The assay uses a human reference gene for sample adequacy and amplification control. All samples positive for coronaviruses using the AusDagnostics assay were further typed using four specific in-house coronavirus type-specific one-step RT-PCRs. Briefly, 5 μl of total nucleic acid was added to 15 μl of reaction mixture using TaqMan Fast Virus 1-Step Master Mix (Thermo Fisher Scientific, Grand Island, NY) and four Custom TaqMan Gene Expression assays (Thermo Fisher Scientific) specific for each of the four human coronaviruses (229E, HKU-1, NL63, and OC43). Cycling conditions were as follows: 5 min at 50°C, 20 s at 95°C, and 40 cycles, with 1 cycle consisting of 3 s at 95°C and 30 s at 60°C. Cycling conditions were universal for all four specific RT-PCRs.

The final composition of the in-house validation panel consisting of 217 clinical samples was 64 SARS-CoV-2 positives, 17 human coronavirus (hCoV) positives (nine NL63 positives, five 229E positives, two HKU-1 positives, and one OC43 positive), 14 human rhinovirus (hRV) positives, nine respiratory syncytial virus (RSV) positives, eight human metapneumovirus (hMPV) positives, eight influenza virus B positives, six influenza virus A (Flu A) positives, and one human parechovirus positive. Four samples each contained multiple respiratory viruses: RSV and hCoV, RSV and hRV, hRV and hCoV-229E, and Flu A and hMPV. Eighty-six samples were negative for all respiratory viruses tested. All samples included in the validation panel were undiluted original 600- μl aliquots of UTM-RT. Aliquots were kept frozen after initial testing at −30°C and thawed 1 h before cobas testing. The median sample storage time at −30°C was 3 days (range, 1 to 17 days). cobas testing of the in-house validation panel was performed in three runs on the afternoon of 24 March 2020.

### Head-to-head prospective comparison.

Prospective cobas evaluation on 502 routinely collected clinical samples against the same comparator (LightMix) was performed in six runs on 25 March 2020 and 26 March 2020. The nasopharyngeal (*n* = 489) or combined nasopharyngeal/oropharyngeal swab (*n* = 13) samples used in prospective head-to-head evaluation were collected in UTM-RT from 502 individuals referred for COVID-19 testing on 25 March 2020 and 26 March 2020. The median transport time of samples from the collection site to the laboratory was 1 h 32 min. Upon arrival in the laboratory, swabs were vortexed for 1 min at maximum speed and two UTM-RT aliquots were prepared: 600 μl for cobas testing and 200 μl for LightMix testing.

### cobas 6800 SARS-CoV-2 testing.

cobas is intended for fully automated sample-to-result qualitative detection of SARS-CoV-2 in nasopharyngeal and oropharyngeal swab samples collected in UTM-RT or Becton, Dickinson Universal Viral Transport System (UVT) from patients with signs and symptoms suggestive of COVID-19. The test could be performed on either the cobas 6800 or cobas 8800 instrument (Roche Molecular Diagnostics, Pleasanton, CA, USA). The cobas 6800 instrument consists of the sample supply module, transfer module, processing module, and analytic module. For detection of SARS-CoV-2, a two-target RT-PCR is used: one targeting ORF1, a nonstructural region that is unique to SARS-CoV-2 (target 1), and the second targeting a conserved region in the structural protein envelope E gene for pan-sarbecovirus detection (target 2). The pan-sarbecovirus primers and probe should also detect the SARS-CoV-2 virus. The test utilizes RNA internal control for sample preparation and PCR amplification process control. Uracil-N-glycosylase is included in the PCR mix to destroy potential contaminating amplicons from previous PCR runs. Automated data management is performed by the manufacturer’s software, which assigns test results for all tests. The results can be reviewed directly on the system screen, printed as a report, or transferred to a laboratory information system. According to the manufacturer’s instructions, a tested sample was considered SARS-CoV-2 positive if cobas showed positive results either for both ORF1 (target 1) and E (target 2) genes or for the ORF1 gene only. In the case of positivity for the E gene only (target 2), the result should be reported as SARS-CoV-2 presumptive positive.

In our study, 600 μl of UTM-RT aliquots equilibrated to room temperature were transferred into barcoded secondary tubes, loaded on the cobas 6800 system, and tested following the manufacturer’s instructions. Testing was performed in batches of 94 samples plus one negative control and one positive control.

### Data analysis.

Contingency tables were constructed to assess overall agreement with 95% confidence intervals (95% CIs), and McNemar’s test was applied to assess differences between matched proportions. The level of agreement between tests was assessed using kappa statistics, and simple linear regression analysis was used for *C_T_* comparative analysis. All statistical analyses were performed using Excel (Microsoft, Redmond, WA, USA) and R software version 3.2.5 (Free Software Foundation, Boston, MA, USA).

## RESULTS

### In-house validation panel.

The results of comparative evaluation of the cobas and LightMix tests on the in-house validation panel consisting of 217 well-characterized samples are summarized in Table 1. Two samples were excluded from analysis due to invalid cobas results. The diagnostic approaches showed overall agreement of 98.1% (211/215; 95% CI, 95.0 to 99.4%), positive agreement of 95.2% (60/63; 95% CI, 85.8 to 98.8%), negative agreement of 99.3% (151/152; 95% CI, 95.8 to 100.0%), and a high kappa value of 0.95 on 215 samples with valid results for both assays. Four discordant results were obtained. Three samples were positive with LightMix and negative by cobas. All three samples with LightMix-positive/cobas-negative results had very low SARS-CoV-2 viral loads with LightMix *C_T_* values for E and RdRp genes of 33.1 and 35.7 (sample 1), 36.5 and 36.9 (sample 2), and 36.9 and 36.0 (sample 3), respectively. Before cobas testing, these three samples with LightMix-positive/cobas-negative results were frozen at −30°C for 12, 5, and 1 day, respectively. Sample 3 was a follow-up/control nasopharyngeal swab of a patient that tested SARS-CoV-2 positive 14 days earlier. As shown in Table 1, one sample (sample 4) gave cobas-positive/LightMix-negative results with cobas *C_T_* values for ORF1 (target 1) and E (target 2) genes of 33.3 and 35.9, respectively.

**TABLE 1:** Results of comparative evaluation of the cobas 6800 SARS-CoV-2 and standardized SARS-CoV-2 detection protocol on validation panel^*a*^

LightMix result	No. of samples with the following result by cobas 6800:			Overall agreement (%) (95% CI)	Kappa value (95% CI)	*P* value by McNemar’s test
	Positive	Negative	Total			
Positive	60	3	63	98.1 (95.0–99.4)	0.95 (0.91–1.00)	0.617
Negative	1	151	152			
Total	61	154	215			

a Results of comparative evaluation of the cobas 6800 SARS-CoV-2 (cobas) and standardized SARS-CoV-2 detection protocol consisting of RNA extraction using the MagNA Pure Compact instrument followed by RT-PCR targeting the E and RdRp coronavirus genes (LightMix) on an in-house validation panel consisting of 217 samples. Two samples were excluded from analysis due to an invalid cobas result. Interpretation of discordant results is given in Results and Discussion.

### Head-to-head prospective comparison.

The results of head-to-head comparative evaluation of the cobas and LightMix tests on 502 samples prospectively collected from the same number of individuals are summarized in Table 2. One sample was excluded from analysis due to an invalid cobas result. The diagnostic approaches showed overall agreement of 99.6% (499/501; 95% CI, 98.4 to 99.9%), positive agreement of 100.0% (63/63; 95% CI:, 92.8 to 100.0%), negative agreement of 99.5% (436/438; 95% CI, 98.2 to 99.9%) and a high kappa value of 0.98 on 501 samples with valid results of both assays. Two discordant results were obtained. One sample (sample 5) with a cobas-positive/LightMix-negative result had a very low SARS-CoV-2 viral load with cobas *C_T_* values for ORF1 (target 1) and E (target 2) genes of 33.8 and 36.5, respectively. According to the cobas manufacturer’s instructions, sample 5 is considered SARS-CoV-2 positive. Sample 5 was a follow-up/control nasopharyngeal swab of a patient that tested SARS-CoV-2 positive using LightMix 15 days earlier. One sample (sample 6) with a cobas presumptive positive/LightMix-negative result had a very low SARS-CoV-2 viral load with cobas *C_T_* values for the E gene (target 2) of 38.1, whereas amplification for the ORF1 gene (target 1) showed a negative result. According to the cobas manufacturer’s instructions, sample 6 was considered SARS-CoV-2 presumptive positive. Sample 6 was also a follow-up/control nasopharyngeal swab obtained from a patient that tested SARS-CoV-2 positive using LightMix 19 days earlier.

**TABLE 2:** Results of prospective head-to-head comparative evaluation of the cobas 6800 SARS-CoV-2 and standardized SARS-CoV-2 detection protocol^*a*^

LightMix result	No. of samples with the following result by cobas 6800:			Overall agreement (%) (95% CI)	Kappa value (95% CI)	*P* value by McNemar’s test
	Positive	Negative	Total			
Positive	63	0	63	99.6 (98.4–99.9)	0.98 (0.96–1.00)	0.480
Negative	2	436	438			
Total	65	436	501			

a Results of prospective head-to-head comparative evaluation of the cobas 6800 SARS-CoV-2 (cobas) and standardized SARS-CoV-2 detection protocol consisting of RNA extraction using the MagNA Pure Compact instrument followed by RT-PCR targeting the E and RdRp coronavirus genes (LightMix) on 502 samples. One sample was excluded from analysis due to an invalid cobas result. Interpretation of discordant results is given in Results and Discussion.

**FIG 1:**
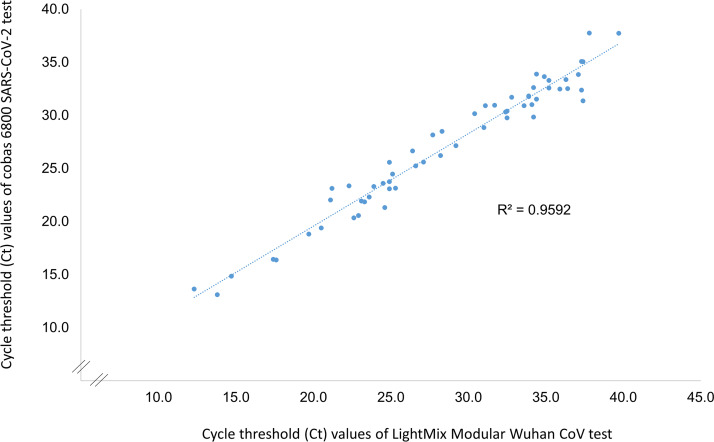
Correlation between cycle threshold (*C_T_*) values obtained by LightMix Modular Wuhan CoV (RdRp gene – SARS-CoV-2-specific target) and cobas 6800 SARS-CoV-2 (target 1 – ORF1 – SARS-CoV-2-specific target) in the prospective head-to-head evaluation performed on 502 samples. *C_T_* values for the LightMix assay were always set at 0.1 normalized reporter dye intensity (delta Rn). Linear regression of the *C_T_* values was performed using samples positive for SARS-CoV-2 by both diagnostic approaches (*n *= 63). The *r*^2^ correlation value is indicated.

As shown in Fig. 1, a remarkably good correlation (*r*^2^ = 0.96) between *C_T_* values for SARS-CoV-2-specific targets obtained by LightMix (RdRp gene) and cobas (ORF1; target 1) was observed on 63 samples positive for SARS-CoV-2 by both diagnostic approaches in the prospective head-to-head evaluation part of our study.

### Additional data supporting the informed decision for a diagnostic approach switch.

After extensive evaluation, our laboratory implemented LightMix-based SARS-CoV-2 testing on 17 January 2020. Routine SARS-CoV-2 testing started on 27 January 2020, and the first positive sample was detected on 4 March 2020 after testing 353 routine samples. As of 8 April 2020, a total of 30,669 SARS-CoV-2 tests have been performed in Slovenia (15,330 tests per million inhabitants), 1,103 laboratory-confirmed cases of COVID-19 have been detected, and 40 deaths have been reported. During a 1-month period before implementation of cobas in routine SARS-CoV-2 testing on 26 March 2020, the workforce needed for SARS-CoV-2 testing using the LightMix approach at our institution gradually increased from a single technician to a total of 14 highly skilled technicians working in two six-member laboratory teams covering 7 a.m. to 11 p.m. shifts plus two technicians for an 8-h night shift. At the time of the diagnostic approach switch, using the LightMix approach, the COVID-19 diagnostic team was able to process 600 to 700 samples per day with an average bench time of 9.6 min per sample. The approximate bench time per sample for cobas testing of 600 to 700 samples per day is around one-tenth of the time needed previously. From 27 March 2020 until 7 April 2020, a total of 2,296 samples have been routinely tested using cobas in 28 runs with a total of 3 samples showing invalid results (1 in 765). All invalid cobas results noticed in our laboratory were caused by either clots/mucus/physical contamination detected by the instrument during sample aspiration or insufficient sample volume identified in sample tubes/processing plates.

## DISCUSSION

In addition to the unprecedented health and economic impact of the COVID-19 pandemic, we are witnessing extraordinary demand on a global scale for personal protective equipment and medical devices such as ventilators as well as sampling devices, reagents, consumables, and diagnostic instruments needed for timely diagnosis of SARS-CoV-2 infection. To meet diagnostic needs and as the pandemic grows, the U.S. FDA recently dramatically expanded enforcement discretion to speed up COVID-19 test access, resulting in granting EUA to 30 different SARS-CoV-2 commercial assays and 5 laboratory developed tests as of 8 April 2020 (https://www.fda.gov/medical-devices/emergency-situations-medical-devices/emergency-use-authorizations#covid19ivd). However, to the best of our knowledge, as of 29 March 2020, no manufacturer-independent evaluation data for any commercial SARS-CoV-2 assay with the U.S. FDA EUA is available in peer-reviewed literature. EUA-granted assays range from those with rapid turnaround time allowing point-of-care testing to those performed on high-throughput diagnostic platforms allowing for large numbers of patients to be tested in a reasonable time frame.

cobas 6800 is a fully automated instrument allowing sample-to-result qualitative and quantitative detection of several microorganisms. A range of cobas 6800 assays have been approved by the U.S. FDA in recent years (https://www.fda.gov/medical-devices/vitro-diagnostics/nucleic-acid-based-tests). Due to the high demand for SARS-CoV-2 testing, problems in further scaling up testing using the LightMix approach, the limited and delayed supply of reagents needed for the LightMix approach, and the availability of cobas 6800 in our laboratory, we decided to evaluate the cobas 6800 SARS-CoV-2 test as soon as it become commercially available. Due to production problems with the cobas 6800/8800 Buffer Negative Control kit, the first reagents arrived in the laboratory with 7-day delay on 24 March 2020. In the meantime, a well-characterized in-house validation panel was prepared and immediately tested with cobas in three runs on the afternoon of 24 March 2020. cobas showed excellent overall agreement with the comparator LightMix on the in-house validation panel with four discordant results (Table 1). All three samples with LightMix-positive/cobas-negative results had very low SARS-CoV-2 viral loads and were most probably cobas negative due to degradation of SARS-CoV-2 RNA during storage/freezing/thawing. Due to the encouraging results obtained on the in-house validation panel, we started immediate prospective head-to-head evaluation of cobas on 502 routinely collected clinical samples against the same comparator. As in the first part of the study, both diagnostic approaches showed excellent overall agreement and a high kappa value also on prospectively collected samples, with only two discordant results (Table 2). Both cobas-positive/LightMix-negative samples had a very low SARS-CoV-2 viral load, suggesting slightly higher analytical sensitivity of cobas over the LightMix approach. On the basis of favorable results obtained in both the retrospective and prospective parts of our study, we decided to start routine SARS-CoV-2 testing with cobas on 26 March 2020. The entire cobas evaluation procedure followed by a successful diagnostic platform switch lasted only 48 h.

Apart from the commercially available cobas 6800 SARS-CoV-2 test that was evaluated in this study, the cobas 6800 instrument can also be used for SARS-CoV-2 detection following a recently published laboratory-developed protocol and the open channel (utility channel) of the cobas 6800 instrument (19). In this study, authors demonstrated good analytical performance of an adapted SARS-CoV-2 assay on swab samples with the limit of detection comparable to that of the LightMix approach used in our evaluation (19). A similar laboratory-developed open-access protocol was also recently published for another U.S. FDA-approved high-throughput instrument, Panther Fusion (Hologic, Marlborough, MA) (20).

In conclusion, the results of the first manufacturer-independent evaluation of cobas performed on a well-characterized in-house validation panel consisting of 217 samples followed by immediate prospective head-to-head comparison on 502 routine samples against the current diagnostic standard showed that cobas is a reliable assay for qualitative detection of SARS-CoV-2 in nasopharyngeal and combined nasopharyngeal/oropharyngeal swab samples collected in the UTM-RT system. Our study showed that, under the extraordinary circumstances that laboratories are currently facing worldwide, a safe diagnostic platform switch is feasible in only 48 h and in the midst of the COVID-19 pandemic, if carefully planned and executed.
